# Appointments Needed for Complete Denture for Frail Older Adults Residing in Long-Term Care Facilities: A Cross-Sectional Study

**DOI:** 10.3390/dj12020036

**Published:** 2024-02-07

**Authors:** Sahr H. Altuwaijri, Tharee Champirat, Chris Wyatt

**Affiliations:** 1 Restorative and Prosthetic Dental Sciences, College of Dentistry, King Saud bin Abdulaziz University for Health Sciences, National Guard Health Affairs, Riyadh 11426, Saudi Arabia; 2 King Abdullah International Medical Research Center, National Guard Health Affairs, Riyadh 11481, Saudi Arabia; 3 Department of Advanced General Dentistry, Faculty of Dentistry, Mahidol University, Bangkok 10400, Thailand; tharee.cha@mahidol.edu; 4 Division of Prosthodontics and Dental Geriatrics, Department of Oral Health Sciences, Faculty of Dentistry, University of British Columbia, Vancouver, BC V6T 1Z4, Canada; cwyatt@dentistry.ubc.ca

**Keywords:** dentures, geriatrics, frailty, long-term care

## Abstract

Frail older adults who reside in long-term care (LTC) facilities face multiple barriers in receiving dental care. In edentulous LTC patients, the fabrication of complete dentures (CDs) can present challenges, leading to an increase in procedural or post-insertion appointments. The aim of this cross-sectional study was to document the number of fabrication and post-insertion follow-up appointments for CDs in frail older adults residing in LTC facilities. Data were collected from electronic patient records (AxiUm) and the Index of Clinical Oral Disorder in Elders (CODE) software utilized by the University of British Columbia Geriatric Dentistry Program from 2002 to 2018. A total of 362 CDs were fabricated between 2002 and 2018 in 272 patients. The mean number of visits required was 4.13 and 4.32, with standard deviations (Std) of 1.45 and 1.25 needed to fabricate maxillary CDs and mandibular CDs, respectively. The mean number of follow-up visits was 1.04 for maxillary dentures and 1.09 for mandibular dentures, with an Std of 1.25 for both, similar to the results obtained for adult patients in community dental clinics. Several factors were found to be associated with an increased number of CD fabrication and follow-up visits. Pre-operative assessment of the patient’s cognitive/physical status and intra-oral condition may indicate the estimated time needed to fabricate CDs.

## 1. Introduction

Canada’s population is aging, with more than 25% of the total population expected to be older than 65 years of age by 2035 [1,2]. Chronic disease and disability associated with aging are linked to poor oral health [3]. Past reports have also suggested that oral disorders affect quality of life and general health and have emphasized the need to improve oral health for this segment of the population [4,5]. Most older adults live in community settings, while approximately 5% of older adults in Canada currently reside in long-term care (LTC) facilities [6,7]. Older adults in Canada are typically admitted to LTC facilities due to complex multimorbidities, advanced cognitive decline, both, or when they can no longer be safely cared for in their own homes or assisted-living residences [7]. This is a vulnerable segment of the population with respect to oral health as this cohort has multiple barriers to accessing dental care [5].

Geriatric patients residing in LTC facilities have high levels of oral disease, including mucosal inflammation, tooth pain, jaw discomfort, and tooth loss [8]. There have been well-documented trends of decreasing edentulism in developed nations over the past 50 years [9]. However, a small cohort of patients still suffer from complete edentulism. In 1990, approximately 17% of the Canadian population was completely edentulous, compared to 6.4% in 2009 [10]. Edentulous patients typically require prostheses to restore oral function and aesthetics, and complete maxillary and mandibular removable dentures are commonly provided to address this need. Edentulous patients typically require five dental appointments to fabricate a complete maxillary or mandibular denture [11]. The entire process includes preliminary impressions, final impressions, jaw records, trying on teeth, and, finally, insertion. Previous studies have reported that, after the delivery of a new prosthesis, one to three post-insertion follow-up visits are typically required for minor adjustments to ensure patient comfort and satisfaction [12,13]. Several simplified complete denture fabrication techniques have also been previously established for elderly patients [13,14,15,16]. With the introduction of digital dentistry in recent years and the use of digital workflows in prosthesis fabrication, complete dentures can be fabricated with fewer appointments [17]. Providing dental treatment to frail older adults residing in LTC facilities is complicated by numerous challenges, including physical and cognitive issues and financial barriers [18]

The aim of this cross-sectional study is to document the number of appointments required to fabricate a complete denture and the number of post-insertion appointments necessary for frail older adults residing in LTC facilities. The study hypothesis is that the number of fabrication visits for a complete denture for frail older adults in LTC facilities will be equal to five visits and that the number of follow-up visits will be between one and three visits, as usually needed for adult patients in community dental clinics.

## 2. Materials and Methods

After human ethics approval (H19-00237) was attained from the University of British Columbia (UBC), data for this study were collected from electronic patient records from the UBC Geriatric Dentistry Program (GDP), including the number of maxillaries and/or mandibular complete removable dentures, the number of fabrication visits, and the number of post-insertion follow-up visits performed for all geriatric patients living in the ten LTC facilities between 2002 to 2018. The UBC GDP has a mandate for clinical service, research, and education in dental geriatrics, with a focus on frail older adults residing in LTC facilities. The data were obtained from the Index of Clinical Oral Disorder in Elders (CODE) software (1.0), which is based on 27 clinical conditions and was previously used in comparative and descriptive research [19], as well as axiUm, a piece of dental management software commonly used in North American dental schools. It can be used for dental charting, managing patients’ appointments, treatment plans, and payments. Data obtained from the CODE software included age, gender, medical conditions, medication, oral hygiene, and mobility. Furthermore, denture-related information, such as opposing arch, mucosal condition, and ridge configuration, was also gathered. The number of visits required for prosthesis fabrication as well as the number of appointments needed for follow-up were identified using axiUm records.

Medical conditions obtained from the CODE software included endocrine diseases such as thyroid disease, diabetes mellitus, etc.; nervous system diseases such as Alzheimer’s disease, dementia, Huntington’s disease, Parkinson’s disease, etc.; behavioral medical conditions such as depression, anxiety, etc.; cardiovascular diseases such as hypertension, etc.; and connective tissue and joint diseases, ENT diseases, diseases of the eye, gastrointestinal conditions, hematological diseases, infectious diseases, kidney and urinary diseases, oncological conditions, respiratory conditions, dermatological conditions, and prosthetic implants. 

The medical conditions were presented in the table as “yes” if the patient presented with the condition and “no” if they did not.

Only the medical conditions that were found to have a relationship with the number of fabrications or follow-up visits are mentioned in the results and tables.

In addition, patient mobility includes categories of ambulatory, wheelchair-bound, or bedridden, and oral hygiene is defined as the patient’s ability to attend to their own oral hygiene (not able to attend to their own oral hygiene, able to brush with assistance, or able to attend to their own oral hygiene).

In the CODE software, the mucosal disorder component includes a score for extreme alveolar atrophy, defined as <1 mm of attached mucosa facially or lingually from the crest of the residual ridge along at least 2 cm of the alveolus, which increases the complexity of fabrication of complete prostheses. A CODE score of 0 reflects no disease, a score of 1 reflects mild disease, a score of 2 reflects moderate disease, and a score of 3 reflects severe disease.

Descriptive analyses including the mean and standard deviation, in addition to frequencies, were used. Chi-squared and Fisher’s exact tests were used to test the differences and associations between the independent factors included in the study.

## 3. Results

**Figure 1:**
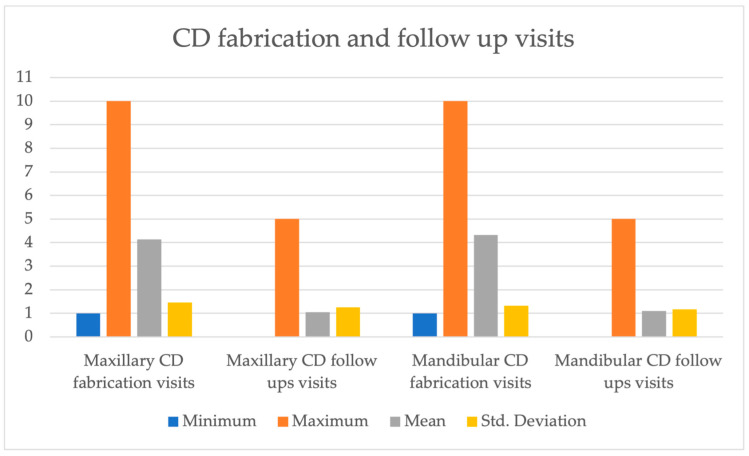
Complete Denture Fabrication and follow up visits.

The study cohort consisted of 154 females and 118 males with a mean age of 82.84 years. A total number of 362 conventional complete dentures were fabricated as part of the UBC GDP between 2002 and 2018. This sample consisted of 181 maxillary complete dentures and 181 mandibular complete dentures. The mean number of visits required to fabricate a maxillary complete denture was 4.13, with an Std of 1.45, while the mean number required to fabricate a mandibular complete denture was 4.32 visits, with an Std of 1.25. The mean number of follow-up visits was 1.04 for maxillary dentures and 1.09 for mandibular dentures, with an Std of 1.25 for both arches, as shown in Figure 1.

### 3.1. Number of Appointments Required for Complete Denture Fabrication

A statistically significant relationship was found between the number of visits needed for mandibular complete denture fabrication and gender. Of the patients who required more than five visits to fabricate mandibular complete dentures, 57.1% were male and 42.9% were female, as shown in Table 1.

In the sub-analysis of medical conditions in the CODE data, a significant relationship was noted between the number of appointments needed to fabricate a maxillary complete denture and a diagnosis of behavioral medical conditions (i.e., depression, anxiety, etc.). Of the patients who required more than five appointments, 62.5% were patients diagnosed with a behavioral condition, while only 37.5% of patients without any behavioral conditions required more than five appointments for the fabrication of a maxillary complete denture, as shown in Table 2.

A diagnosis of endocrine disease was also found to influence the number of appointments needed for maxillary complete denture fabrication. Of the patients who required more than five appointments, 6.3% were found to be patients with endocrine medical conditions, while 93.8% were patients without endocrine medical conditions, as shown in Table 2. All of the medical conditions included in our study had no effect on the number of mandibular complete denture fabrication appointments.

The analysis of mucosal disorder scores performed using the CODE data revealed a relationship between CODE scores for mucosal disease and the number of appointments required for both maxillary and mandibular complete denture fabrication. Approximately 37.5% of patients who required more than five appointments for maxillary complete denture fabrication were patients with a good mucosal condition, while the rest of the patients who required more than five appointments were patients with moderate and severe mucosal disorders, as shown in Table 2. For mandibular complete denture fabrication, 40% of patients who required more than five appointments for the fabrication of a mandibular complete denture were patients with a good mucosal condition, while the other 60% of patients included 30% of patients with a moderate mucosal condition and 30% of patients with a severe mucosal disorder, as shown in Table 1.

Significant differences were found in the number of appointments required for complete denture fabrication when both maxillary and mandibular complete dentures were made at the same time compared to when only one prosthesis was being made. Overall, more appointments were required when both maxillary and mandibular dentures were being fabricated concurrently. Approximately 25% of the patients who required more than five appointments were patients who needed single maxillary complete dentures, compared to the majority of patients (75%) with a complete set of dentures, as shown in Table 2. Of the patients who required more than five appointments, 33.3% were patients with single mandibular complete dentures, while 66.7% were patients with a complete set of dentures, as shown in Table 1.

### 3.2. Number of Appointments Required for Post-Insertion Follow-Up

The results of the study show a significant difference between the type of treatment (i.e., a single denture or complete set of dentures) and the number of post-insertion follow-up appointments. The majority 69.23% of the patients who needed 4–5 post-insertion visits were patients with a complete set of dentures, while 30.77% of the appointments were for patients with a single maxillary denture, as shown in Table 3. For mandibular complete denture follow-up appointments, 10% of patients who attended 4–5 post-insertion appointments were found to be patients with single mandibular complete dentures, which was significantly different from the 90% of patients that took 4–5 appointments, who were the patients that had complete sets of dentures, as shown in Table 4.

The mobility of patients (i.e., ambulatory, wheelchair-bound, or bedridden) had a significant effect on the number of post-insertion follow-up visits for both maxillary and mandibular complete dentures. For patients requiring 4–5 post-insertion follow-up appointments after the fabrication of a maxillary complete denture, 92.31% were wheelchair-bound, 7.69% were bedridden, and none of them were ambulatory, as shown in Table 3. Similarly, for patients requiring 4–5 post-insertion follow-up visits after the fabrication of a mandibular complete denture, 90% were wheelchair-bound, 10% were bedridden, and none of them were ambulatory, as shown in Table 4.

Most medical conditions did not have a significant influence on the number of post-insertion follow-up appointments for both maxillary and mandibular complete dentures. However, patients with endocrine conditions showed a significant difference in the number of maxillary complete denture follow-up visits. Approximately 92.31% of the patients who needed 4–5 appointments were patients without endocrine disease, while 7.69% were patients diagnosed with endocrine conditions, as shown in Table 3.

A patient’s ability to attend to their own oral hygiene also had a significant effect on the number of post-insertion follow-up visits for both maxillary and mandibular complete dentures. Approximately 53.85% of patients who required 4–5 follow-up appointments for maxillary complete dentures were found to be patients who were not able to attend to their own oral hygiene, while 23.08% comprised patients who could brush with assistance. Patients who are able to attend to their own oral hygiene comprised 23.07% of the patients requiring 4–5 follow-up appointments, as shown in Table 3. For mandibular complete dentures, patients who could not attend to their own oral hygiene constituted 70% of the patients who required 4–5 follow-up appointments, while 20% were patients who could attend to their own oral hygiene with assistance. The last 10% comprised patients who could attend to their own oral hygiene, as shown in Table 4.

## 4. Discussion

The study results show that the mean numbers of visits for maxillary and mandibular complete dentures were 4.13 and 4.32, respectively, which were around the five visits required for adult patients in community dental clinics. Several factors were found to affect the number of fabrication visits, including gender, behavioral conditions, endocrine conditions, mucosal disorders, and single dentures vs. complete sets of dentures. The mean number of fabrication visits could be explained by the fact that the treatment may have been modified by eliminating one or more of the steps, or the fact that some procedures were combined by the treating dentist to accommodate the patient’s medical condition. The mean numbers of follow-up visits were 1.04 for maxillary dentures and 1.09 for mandibular dentures. The number of follow-up visits was found to be affected by the presence of endocrine conditions, the mobility of the patient, the ability to attend to personal OH, and the type of treatment—whether it involved a single denture or a complete set of dentures.

### 4.1. Number of Appointments Required for Complete Denture Fabrication

In this study, gender was one of the factors that was found to be related to the number of visits needed for mandibular complete denture fabrication. Male patients required more appointments for the fabrication of mandibular complete dentures than females. This may be explained by the fact that the mandibular arch is affected by the surrounding muscle strength and bite forces compared to the maxillary arch. That being said, males are known to have higher bite forces and muscle thickness and strength, which may have contributed to our study results. Muscle strength can act as an influencing factor when impressions are taken, as well as during the jaw record process [20,21]. Patients with moderate and severe mucosal disorders required more appointments for the fabrication of maxillary and mandibular complete dentures. This is due to the increased difficulty of handling these conditions, including ridge atrophy, ridge fibrosis, and mucosal hyperplasia, and, in particular, difficulties in capturing the residual ridge when impressions are taken, and several studies have suggested different techniques in order to facilitate the procedure and thus provide a stable record basis for the processes of jaw records and the patient trying on the teeth [22]. It was found that patients with behavioral and psychiatric disorders, including anxiety, depression, schizophrenia, major affective disorders, alcoholism, sleep disorders, and seizures, required more appointments to fabricate maxillary complete dentures than patients without behavioral disorders. This can be explained by the fact that people with such disorders usually exhibit irregular attendance of dental appointments or the avoidance of dental treatment [23]. Another factor identified as influencing the number of visits is endocrine diseases, including diabetes and hypothyroidism. One reason as to why only one patient (6.3%) with an endocrine medical condition required more than five appointments for maxillary complete denture fabrication is the fact that diabetes causes low salivary flow, which influences the ease with which each step can be carried out, as the dryness of the mucosa and the palate can increase the difficulty of carrying out some of the steps and make the procedure uncomfortable for the patient [24]. Lastly, for complete denture fabrication appointments, we found that fabricating a complete set of maxillary and mandibular dentures required more appointments than the fabrication of a single maxillary or mandibular complete denture. This could be due to each procedure being performed for both arches, which results in more steps and more time being required, thus leading to additional appointments.

### 4.2. Number of Appointments Required for Post-Insertion Follow-Up

In addition, for the post-insertion visits, we found that the fabrication of a complete set of dentures required more appointments due to there being more work required to adjust both dentures. Other factors that affected the number of follow-ups were the degree of patient mobility and oral hygiene. Patients who were wheelchair-bound and bedridden were the only patients that required more than three post-insertion follow-ups for both maxillary and mandibular dentures. This can be justified by the increased difficulty of performing the clinical steps in both procedures, which thus leads to more time being required and, eventually, more appointments. Patients who presented with difficulty in attending to their oral hygiene represented the majority of the patients who required more than three follow-up appointments, which might be explained by the fact that they are at higher risk of post-insertion complications such as denture stomatitis due to their compromised oral hygiene [25].

### 4.3. Strengths and Limitations

The current study examined the number of visits needed for the fabrication of complete dentures and follow-up appointments in frail elderly patients residing in long-term care facilities in Vancouver, Canada, between 2022 and 2018. The study sample size was robust, with several possible related factors being assessed to provide good, reliable data that can be used in the future. Dental practitioners treating frail elderly patients can use these data to predict the number of appointments required and the ease or difficulty of certain treatments after assessing the patient’s medical condition(s). That being said, however, this is a retrospective study using a single data source in one community to assess multiple factors, which is helpful in terms of finding out any related factors and at the same time increases the complexity of the study. Further retrospective or prospective clinical studies assessing similar or different factors in other communities would be helpful in applying the conclusions to a wider range of populations.

## 5. Conclusions

The results of this study show that the mean numbers of visits required for maxillary and mandibular complete denture fabrication were 4.13 and 4.32, respectively, in elderly patients residing at LTC facilities, which were around the 5 visits needed for adult patients in community dental clinics. The mean number of follow-up visits was found to be 1.04 for maxillary dentures and 1.09 for mandibular dentures. This was similar to the number of visits needed for post-insertion adjustment appointments. Further studies are recommended to explore other possible factors and associations related to an increased number of appointments.

## Figures and Tables

**Table 1:** Mandibular CD Fabrication Visits.

Mandibular CD Fabrication Visits					
	<5 Visits	5 visits	>5 Visits	Total	Chi-Squared Tests of Independence
Gender					Fisher’s Exact Test
Female	62 (66.7%)	34 (51.5%)	9 (42.9%)	105 (58.3%)	0.048
Male	31 (33.3%)	32 (48.5%)	12 (57.1%)	75 (41.7%)	
Total	93 (100%)	66 (100%)	21 (100%)	180 (100%)	
Mucosal conditions					Fisher’s Exact Test
Good	74 (79.6%)	49 (74.2%)	8 (40.0%)	131 (73.2%)	0.006
Moderate	11 (11.8%)	12 (18.2%)	6 (30.0%)	29 (16.2%)	
Severe	8 (8.6%)	5 (7.6%)	6 (30.0%)	19 (10.6%)	
Total	93 (100%)	66 (100%)	20 (100%)	179 (100%)	
Single vs. complete set					Fisher’s Exact Test
Single denture	61 (64.9%)	24 (36.4%)	7 (33.3%)	92 (50.8%)	0.000
Complete set	33 (35.1%)	42 (63.6%)	14 (66.7%)	89 (49.2%)	
Total	94 (100%)	66 (100%)	21 (100%)	181 (100%)	

**Table 2:** Maxillary CD Fabrication Visits.

Maxillary CD Fabrication Visits					
	<5 Visits	5 Visits	>5 Visits	Total	Chi-Squared Tests of Independence
Behavioral Group					Fisher’s Exact Test
No	69 (71.1%)	47 (69.1%)	6 (37.5%)	122 (67.4%)	0.033
Yes	28 (28.9%)	21 (30.9%)	10 (62.5%)	59 (32.6%)	
Total	97 (100%)	68 (100%)	16 (100%)	181 (100%)	
Endocrine Group					Fisher’s Exact Test
No	61 (62.9%)	47 (69.1%)	15 (93.8%)	123 (68.0%)	0.039
Yes	36 (37.1%)	21 (30.9%)	1 (6.3%)	58 (32.0%)	
Total	97 (100%)	68 (100%)	16 (100%)	181 (100%)	
Mucosal conditions					Fisher’s Exact Test
Good	83 (86.5%)	52 (77.6%)	6 (37.5%)	141 (78.8%)	0.001
Moderate	5 (5.2%)	9 (13.4%)	5 (31.3%)	19 (10.6%)	
Severe	8 (8.3%)	6 (9.0%)	5 (31.3%)	19 (10.6%)	
Total	96 (100%)	67 (100%)	16 (100%)	179 (100%)	
Single vs. complete set					Fisher’s Exact Test
Single denture	64 (66.0%)	24 (35.3%)	4 (25.0%)	92 (50.8%)	0.000
Complete set	33 (34.0%)	44 (64.7%)	12 (75.0%)	89 (49.2%)	
Total	97 (100%)	68 (100%)	16 (100%)	181 (100%)	

**Table 3:** Maxillary CD Post-Insertion Follow ups.

Maxillary CD Post-Insertion Follow Ups					
	No Post-Insertion Follow-Up	1–3 Post-Insertion Follow-Ups	4–5 Post-Insertion Follow-Ups	Total	Chi-Squared Tests of Independence
OH Group					Fisher’s Exact Test
Yes	43 (58.90%)	41 (49.39%)	3 (23.07%)	87 (51.48%)	0.027
No	12 (16.44%)	26 (31.33%)	7 (53.85%)	45 (26.63%)	
With assistance	18 (24.66%)	16 (19.28%)	3 (23.08%)	37 (21.89%)	
Total	73 (100%)	83 (100%)	13 (100%)	169 (100%)	
Endocrine Group					Fisher’s Exact Test
No	45 (56.25%)	66 (75%)	12 (92.31%)	123 (67.96%)	0.005
Yes	35 (43.75%)	22 (25%)	1 (7.69%)	58 (32.04%)	
Total	80 (100%)	88 (100%)	13 (100%)	181 (100%)	
Mobility					Fisher’s Exact Test
Ambulatory	39 (49.37%)	34 (39.53%)	0 (0.0%)	73 (41.01%)	0.004
Wheelchair-bound	37 (46.84%)	45 (52.33%)	12 (92.31%)	94 (52.81%)	
Bedridden	3 (3.79%)	7 (8.14%)	1 (7.69%)	11 (6.18%)	
Total	79 (100%)	86 (100%)	13 (100%)	178 (100%)	
Single vs. complete set					Fisher’s Exact Test
Single denture	52 (65%)	36 (40.91%)	4 (30.77%)	92 (50.83%)	0.002
Complete set	28 (35%)	52 (59.09%)	9 (69.23%)	89 (49.17%)	
Total	80 (100%)	88 (100%)	13 (100%)	181 (100%)	

**Table 4:** Mandibular CD Post-Insertion Follow-Ups.

Mandibular CD Post-Insertion Follow-Ups					
	No Post-Insertion Follow-Up	1–3 Post-Insertion Follow-Ups	4–5 Post-Insertion Follow-Ups	Total	Chi-Squared Tests of Independence
OH Group					Fisher’s Exact Test
Yes	38 (58.46%)	55 (57.29%)	1 (10%)	94 (54.97%)	0.010
No	13 (20%)	27 (28.13%)	7 (70%)	47 (27.49%)	
With assistance	14 (21.54%)	14 (14.58%)	2 (20%)	30 (17.54%)	
Total	65 (100%)	96 (100%)	10 (100%)	171 (100%)	
Mobility					Fisher’s Exact Test
Ambulatory	35 (51.47%)	40 (40.40%)	0 (0.0%)	75 (42.37%)	0.014
Wheelchair-bound	30 (44.12%)	50 (50.51%)	9 (90%)	89 (50.28%)	
Bedridden	3 (4.41%)	9 (9.09%)	1 (10%)	13 (7.35%)	
Total	68 (100%)	99 (100%)	10 (100%)	177 (100%)	
Single vs. complete set					Fisher’s Exact Test
Single denture	40 (58.82%)	51 (49.51%)	1 (10%)	92 (50.83%)	0.012
Complete set	28 (41.18%)	52 (50.49%)	9 (90%)	89 (49.17%)	
Total	68 (100%)	103 (100%)	10 (100%)	181 (100%)	

## Data Availability

The datasets used and analyzed during the current study are available at UBC.
